# Predicting multiple sclerosis severity with multimodal deep neural networks

**DOI:** 10.1186/s12911-023-02354-6

**Published:** 2023-11-09

**Authors:** Kai Zhang, John A. Lincoln, Xiaoqian Jiang, Elmer V. Bernstam, Shayan Shams

**Affiliations:** 1 Department of Health Data Science and Artificial Intelligence, McWilliams School of Biomedical Informatics, University of Texas Health Sciences Center at Houston, Houston, TX USA; 2 Department of Neurology, University of Texas Health Sciences Center, McGovern Medical School, Houston, TX USA; 3 Division of General Internal Medicine, Department of Internal Medicine, University of Texas Health Sciences Center, McGovern Medical School, Houston, TX USA; 4 Department of Applied Data Science, San Jose State University, San Jose, CA USA

**Keywords:** Multimodal deep learning, Multiple sclerosis, Expanded disability status scale

## Abstract

Multiple Sclerosis (MS) is a chronic disease developed in the human brain and spinal cord, which can cause permanent damage or deterioration of the nerves. The severity of MS disease is monitored by the Expanded Disability Status Scale, composed of several functional sub-scores. Early and accurate classification of MS disease severity is critical for slowing down or preventing disease progression via applying early therapeutic intervention strategies. Recent advances in deep learning and the wide use of Electronic Health Records (EHR) create opportunities to apply data-driven and predictive modeling tools for this goal. Previous studies focusing on using single-modal machine learning and deep learning algorithms were limited in terms of prediction accuracy due to data insufficiency or model simplicity. In this paper, we proposed the idea of using patients’ multimodal longitudinal and longitudinal EHR data to predict multiple sclerosis disease severity in the future. Our contribution has two main facets. First, we describe a pioneering effort to integrate structured EHR data, neuroimaging data and clinical notes to build a multi-modal deep learning framework to predict patient’s MS severity. The proposed pipeline demonstrates up to 19% increase in terms of the area under the Area Under the Receiver Operating Characteristic curve (AUROC) compared to models using single-modal data. Second, the study also provides valuable insights regarding the amount useful signal embedded in each data modality with respect to MS disease prediction, which may improve data collection processes.

**Supplementary Information:**

The online version contains supplementary material available at 10.1186/s12911-023-02354-6.

## Introduction

Multiple sclerosis (MS) is a neurodegenerative condition characterized by potential disability, affecting the central nervous system comprising the brain and spinal cord. Estimations based on a ten-year accumulation up until 2010 reveal a prevalence of over 700,000 cases of MS in adult individuals within the United States [1]. Recent advancements in MS research have unveiled a significant neuron count loss of up to 39% in patients who succumbed to MS compared to those unaffected by the disease [2]. Although the human brain possesses inherent self-repair mechanisms and regenerative potential capable of addressing brain plaques [3], the extent of such abilities remains notably limited. Hence, timely intervention to prevent or decelerate brain damage assumes critical importance in MS treatment [4]. Accurate grading of MS severity plays a vital role in determining effective treatment approaches, with scoring systems widely employed for this purpose. One such commonly employed ordinal scoring system is the EDSS [5], frequently utilized by healthcare providers to assess clinical disability in MS. This comprehensive scale encompasses diverse functional systems, including pyramidal functions (muscle strength, tone, and reflexes), cerebellar functions (coordination and balance), brainstem functions (eye movements, speech, and swallowing), sensory functions (light touch, pain, and vibratory sense), bowel and bladder functions, visual functions, cerebral functions (cognition), and ambulation. Building upon the EDSS, Roxburgh et al. proposed the Multiple Sclerosis Severity Score, facilitating the determination of MS disease progression using single assessment data, particularly in cases where only one evaluation is available throughout the course of the disease [6]. Several milestones defined within the EDSS score have commonly been adopted to delineate different stages of the MS disease course. The EDSS 4 (significant disability but capable of walking without aid or rest for 500 m), EDSS 6 (requires unilateral assistance to walk approximately 100 m with or without resting), and EDSS 7 (ability to walk no more than 10 m without rest while relying on support from a wall or furniture) serve as notable milestones frequently employed in the study of MS disease severity.

The evaluation of a patient’s EDSS score requires the expertise of a well-trained specialist to ensure accurate assessment, which limits its applicability to clinics specialized in MS disease. Several research studies have endeavored to tackle this challenge by employing machine learning or deep learning models. For instance, Pinto et al. proposed the utilization of machine learning models to predict MS progression based on the clinical characteristics observed during the initial five years of the disease [7]. Zhao et al. employed a support vector machine (SVM) classifier along with demographic, clinical, and MRI data from the first two years to forecast patients’ EDSS scores at five-year follow-ups [8]. Sacca et al. explored various machine learning models, such as Random Forest, Support Vector Machine, NaiveBayes, K-nearest-neighbor, and Artificial Neural Network, and employed functional MRI-derived features to classify MS disease severity [9]. Narayana et al. proposed the adoption of the VGG-16 convolutional neural network (CNN) to predict enhancing lesions in MS patients using non-contrast MRIs [10]. D’Costa et al. introduced a transformer model named MS-BERT to predict EDSS scores based on patients’ neurological consultation notes [11]. Ciotti devised a clinical instrument to retrospectively capture EDSS levels, achieving a Kappa score of 0.80 when comparing captured EDSS scores with actual values [12]. Chase et al. also utilized neurological consultation notes, employing simpler models (Naïve Bayes classification model) and features (word frequency) [13]. Dekker et al. employed multiple linear regression models on patient brain lesion volumes and their variations over time to predict physical disability [14].

The above studies explored the application of machine learning and deep learning methods, however, they predominantly focused on limited single modality patient information (such as clinical notes, basic lesion volume information extracted from MRI, or patient clinical characteristics). In recent years, the field of multimodal deep learning has witnessed significant advancements. These advancements primarily revolve around three key research questions: addressing modality heterogeneity, identifying interconnections between modalities, and representing their interactions effectively [15]. Based upon the recent advancements in multimodal deep learning, it is reasonable to posit that leveraging multimodal deep learning approaches can integrate fragmented information from diverse modalities, leading to more accurate predictions of MS disease severity. Hence, this study endeavors to address the question of whether harmonizing the available EHR data modalities collected during patient clinic visits and leveraging longitudinal data can enable more precise prediction of MS severity. We investigate the potential of utilizing patients’ MRI images, clinical notes, and structured EHR data, encompassing laboratory tests, vital sign observations, medication prescriptions, and patient demographics, collected during clinic visits, to predict MS disease severity three years ahead.

We propose a multimodal deep neural network architecture capable of leveraging diverse modalities within MS patient EHR data. This includes MRI images, such as pre- and post-contrast T1 weighted images, T2 weighted images, fluid-attenuated inversion recovery images, and proton density images. By harnessing this comprehensive set of modalities, our approach aims to achieve accurate prediction of MS disease severity. In addition, we propose the utilization of patients’ longitudinal data for predicting EDSS milestones. This approach acknowledges that evidence regarding patients’ MS disease severity is not solely confined to the most recent EHR data but is also abundantly present in the data from previous clinic visits. By incorporating both the current clinic visit and historical EHR data, our proposed multimodal deep neural network surpasses the limitations of using solely cross-sectional data (e.g., utilizing clinical notes from the current visit to predict EDSS scores [11]). Longitudinal data encompasses a wealth of MS disease progression information, surpassing that of cross-sectional data, thereby enhancing the model’s ability to generate more accurate predictions of the patient’s future status.

This study makes four key contributions.

- A novel deep learning architecture, namely a multimodal neural network, coupled with a data fusion mechanism. This architecture efficiently incorporates diverse EHR components, including medications, vital signs, laboratory test results, clinical imaging, and physician notes, to address the challenging task of predicting MS disease severity. The experimental results demonstrate noteworthy enhancements in prediction accuracy when compared to using single modality data or simpler models.
- Utilization of longitudinal data, encompassing both current and historical visit information, instead of relying solely on cross-sectional data from the current visit. This approach enables precise classification of patient EDSS score milestones during the current clinic visit.
- Exploration of the informative content embedded within each data modality for MS severity prediction. The proposed neural network employs various attention mechanisms to enhance both prediction accuracy and model explainability. These mechanisms provide insights into the importance of different data modalities, thereby shedding light on the specific aspects contributing significantly to the prediction process.
- We have developed an end-to-end AI model designed to work efficiently with readily accessible data, significantly reducing the necessity for complex preprocessing procedures. In contrast to methods that demand intricate feature extraction steps, such as the measurement of thalamic volume or lateral ventricle volume, our proposed model streamlines the preprocessing stage. It achieves this by leveraging deep learning to autonomously discover features and interactions, simplifying the training process while preserving strong predictive performance.

## Materials and methods

In this section, we provide an overview of the patients’ data descriptions, our neural network architecture, and our innovative techniques for addressing the common issues in multiple data modality modeling, such as missing data, irregular sampling, data fusion.

### Data description

Our database comprises a comprehensive dataset of 300 patients diagnosed with MS. Table 1 provides a summary of the demographic information of these patients. Each patient’s data encompasses three distinct modalities: (1) neuroimaging data, (2) structured EHR data, and (3) clinical notes.

**Table 1:** An overview of patient statistics in the dataset (SD: standard deviation)

		Average $\pm$ SD	Minimum	Maximum	0.25 quantile	0.75 quantile
Age		43.62 $\pm$ 11.20	19.00	71.00	34.00	52.00
EDSS @ baseline		1.93 $\pm$ 1.59	0	7.50	1.00	2.50
EDSS @ last visit		2.90 $\pm$ 1.96	0	9.50	1.50	3.50
Number of visits		3.39 $\pm$ 1.60	1	13.00	2.00	4.00
Follow-up years (Years b/w first and last visits)		5.14 $\pm$ 4.34	0	22.66	2.03	7.01
Number of MRI sessions/patient		1.20 $\pm$ 0.96	0	4	0	2
Gender	Male	66 (22.0%)				
	Female	234 (78.0%)				
Race	White	197 (65.67%)				
	Black or African American	103 (34.33%)				
Ethnicity	Hispanic or Latino	100 (33.33%)				
	NOT Hispanic or Latino	200 (66.67%)				

The neuroimaging data is stored in NIFTI format and captures the patients’ brain images. Most patients have undergone multiple clinical visits, and during each visit, a range of information is recorded in the structured EHR data. This includes laboratory test results, vital signs, prescribed medications, diagnoses, medical procedures, and treatments, which are stored in separate tables.

The clinical notes consist of detailed descriptions provided by physicians during each clinic visit, offering valuable insights into the patient’s condition. Our proposed neural network architecture is specifically designed to handle the heterogeneous structure of these databases by learning representations from each modality.

The prediction objective of this research is focused on a classification problem, aiming to predict whether a patient will reach specific milestones on the EDSS with a specified time frame, particularly three years in advance. For all 300 patients, the EDSS was evaluated and recorded by physicians at the end of each clinic visit, and these scores serve as the ground truth labels. For patients with a follow-up time (i.e., the time interval between the first and last clinic visit) of less than three years, we utilize their data from the first clinic visit to predict the score at the last visit.

**Fig. 1:**
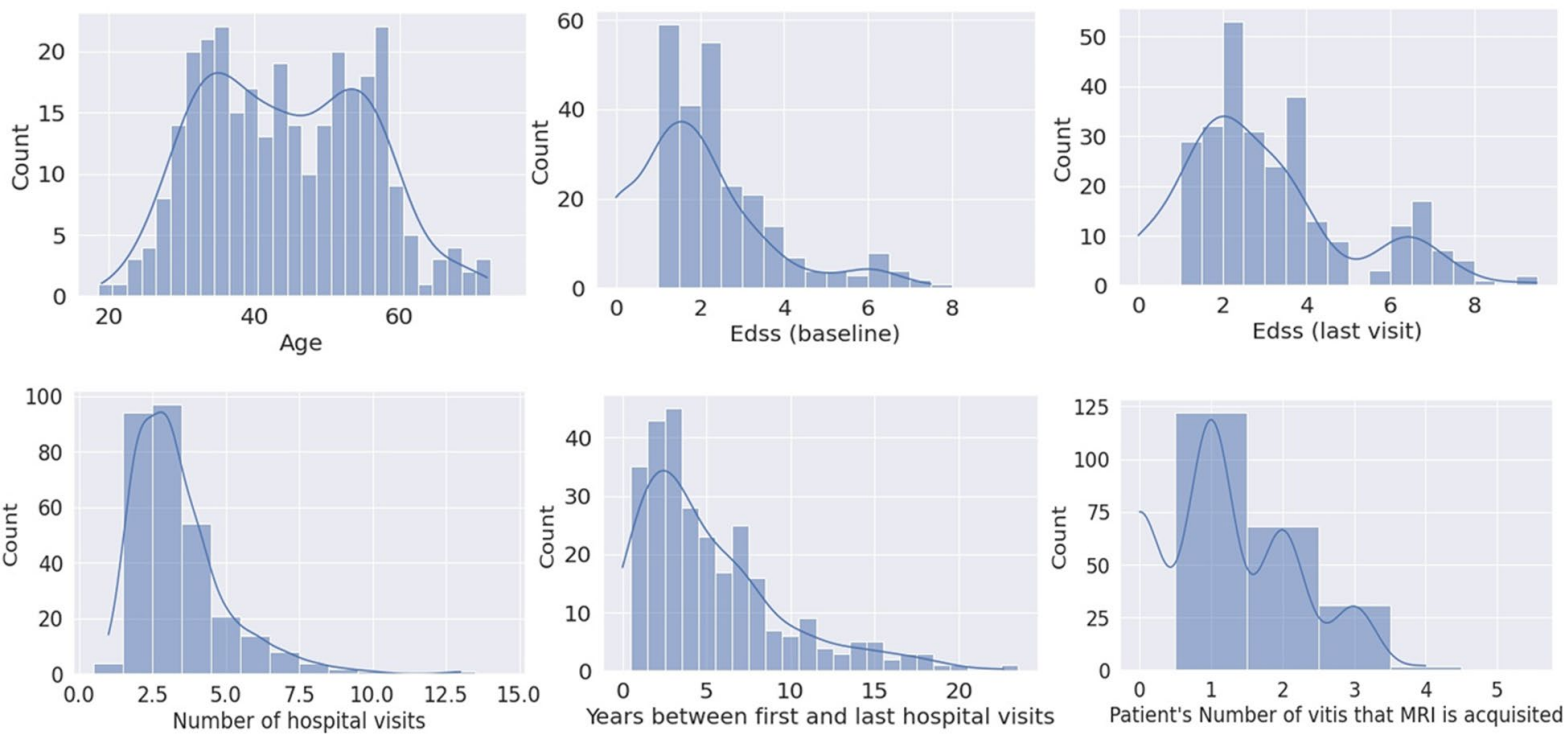
The histograms of all patients by age; baseline EDSS (at initial hospital visit); EDSS at the last hospital visit; total hospital visits; years between the first and the last hospital visit; number of hospital visits during which brain MRI scan was performed

**Fig. 2:**
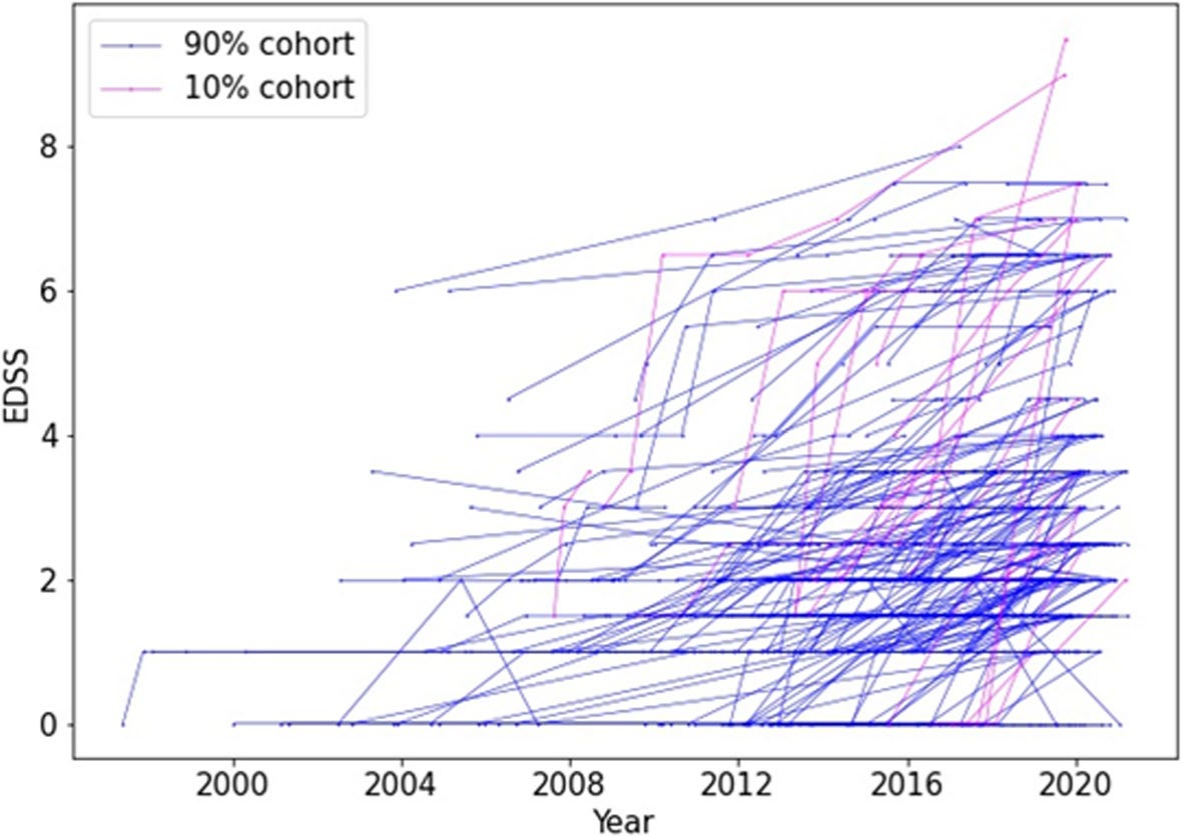
The MS disease progression of all patients. For clear illustration, patient were sorted by the total EDSS increase in their disease course and the trajectory of the top 10% cohort who grows the most were marked in red, and the rest 90% cohort were marked

Figure 1 illustrates the distributions of patients’ ages and EDSS scores. Additionally, Fig. 2 presents the EDSS historical scores of all patients over the course of their disease, offering insights into the progression of their condition.

#### Brain MRI

A total of 360 MRI images were obtained for the 300 patients included in the study. The imaging studies were conducted using a Philips 3.0T Ingenia scanner (Philips Medical Systems, Best, Netherlands). Multiple MRIs were available for some patients, collected from different clinic visits. The MRI dataset encompasses five distinct sequences: pre-contrast and post-contrast T1-weighted sequences (T1-pre, T1-post), T2-weighted sequences, proton density-weighted sequences (PD), and fluid-attenuated inversion recovery sequences (FLAIR).

**Fig. 3:**
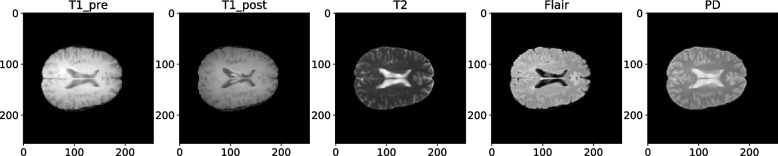
The MRI sequences of a patient as an example

All MRI sequences were acquired with a field of view of 256 mm x 256 mm x 44 mm and in the axial plane. To ensure consistency and facilitate analysis, the MRI images underwent several preprocessing steps. First, they were skull-stripped using the Simple Skull Stripping (S3) method [16] and the SRI24 template [17]. Next, a bias correction technique known as N4 Bias Field Correction was applied to adjust the low-frequency intensity variations [18]. Finally, the images were co-registered to a common template (SRI24) using FreeSurfer [19]. A representative example of the MRI sequences for a sample patient is displayed in Fig. 3. These processed MRI images serve as a crucial component of the multimodal dataset, contributing valuable information for the subsequent analysis and prediction tasks.

#### Clinical notes

The patient’s clinical notes are documented in unstructured free-text format and provide a comprehensive account of the patient’s health status. These notes encompass a range of vital information, including the physician’s observations, patient demographics (such as weight, height, and BMI - body mass index), physiological condition, medical diagnoses, prescribed medications, and administered treatments. To ensure privacy and confidentiality, all clinical notes data underwent a rigorous de-identification process, where any personally identifiable information of both patients and physicians was removed from the dataset. This approach adheres to stringent privacy regulations and safeguards the anonymity of the individuals involved, allowing for secure and ethical analysis of clinical data.

#### Structured EHR

The patient’s structured EHR consists of organized tabular data that encompasses various types of information, including laboratory test measurements (floating-point values), vital sign observations (floating-point values), medication administrations (binary indicator − 0 for not taken, 1 for taken), and demographic information (age: floating-point value, race/ethnicity/gender: binary indicators). The EHR tables are constructed in a standardized format, where each row represents an observational time stamp, and the columns represent specific features. It is important to note that the features within each table remain consistent for all patients, while the number of rows may vary depending on the number of observational time points for each patient.

To streamline the EHR tables and facilitate effective neural network training, we apply a time granularity of 4 h for laboratory tests, vital signs, and medication tables. During each 4-hour window, we calculate the average value for each feature if multiple observations are available. This approach serves to reduce table dimensions, eliminate observational noise, and prevent the creation of large and sparse tables that could hinder neural network training. When certain features lack observations within the 4-hour window, the corresponding entry is set to zero.

It is important to maintain the integrity of the data within clinic encounters, ensuring that each 4-hour window falls within a single encounter. This prevents the averaging of feature values from different encounters. For example, if a patient has two clinic encounters, one from 2014-05-05 1:15:00 PM to 2014-05-05 6:00:00 PM, and another from 2015-09-20 9:12:00 AM to 2015-09-20 1:00:00 PM, there would be four rows in each table representing the observations from specific time intervals within each encounter. Rows containing all zeros (indicating no observations for any feature) are deleted. The demographic data of all patients is structured as a fixed size vector, providing a standardized representation of the demographic variables. Table 2 presents the variables utilized in our dataset.

**Table 2:** The features from the structured EHR data tables, including laboratory tests, vital signs, and medications

LABORATORY TEST			VITAL SIGN	MEDICATION
Mean Corpuscular Hemoglobi	Carbon Dioxide	Albumin	Diastolic Blood Pressure	Baclofen
Red Cell Distribution Width	Basophils	Glucose Level	Systolic Blood Pressure	Gabapentin
Mean Corpuscular Hemoglobin Concentration	White Blood Cell Count	eGFR	Heart Rate	Copaxone
Mean Corpuscular Volume	Hematocrit	Albumin/Globulin Ratio	Weight	Gilenya
Alanine Aminotransferase	Red Blood Cell Count	Eosinophils	Height	Tecfidera
Aspartate Aminotransferase	Platelet Count	Potassium Level	BMI	Aubagio
Anion Gap	Total Protein	Creatinine	O2 Saturation	Ampyra
MRI Brain W/Wo Contrast	Bili Total	Bilirubin, Direct	Pulse	Prednisone
Creatinine Level	Alkaline Phosphatase	Bun/Creatinine Ratio	Temperature	Vitamin
Bun/Creatinine Ratio	Albumin Level	Potassium	Respiration	Duloxetine
Hematocrit Test	Globulin	Systolic		Dalfampridine
Hemoglobin	Neutrophils	MRI Spine Cervical W Wo Contrast		Clonazepam
Blood Urea Nitrogen	Lymphocytes	Brain W/Wo Contrast MRI		
Mean Platelet Volume	Absolute Eosinophils	Body Surface Area		
Calcium Level Total	Basophils	Bilirubin, Indirect		
Sodium Level	Absolute Monocytes	Segmented Neutrophils		
Thyroid Stimulating Hormone	Absolute Neutrophils	Monocytes		
Segs-Bands	Absolute Basophils	Chloride Level		

**Fig. 4:**
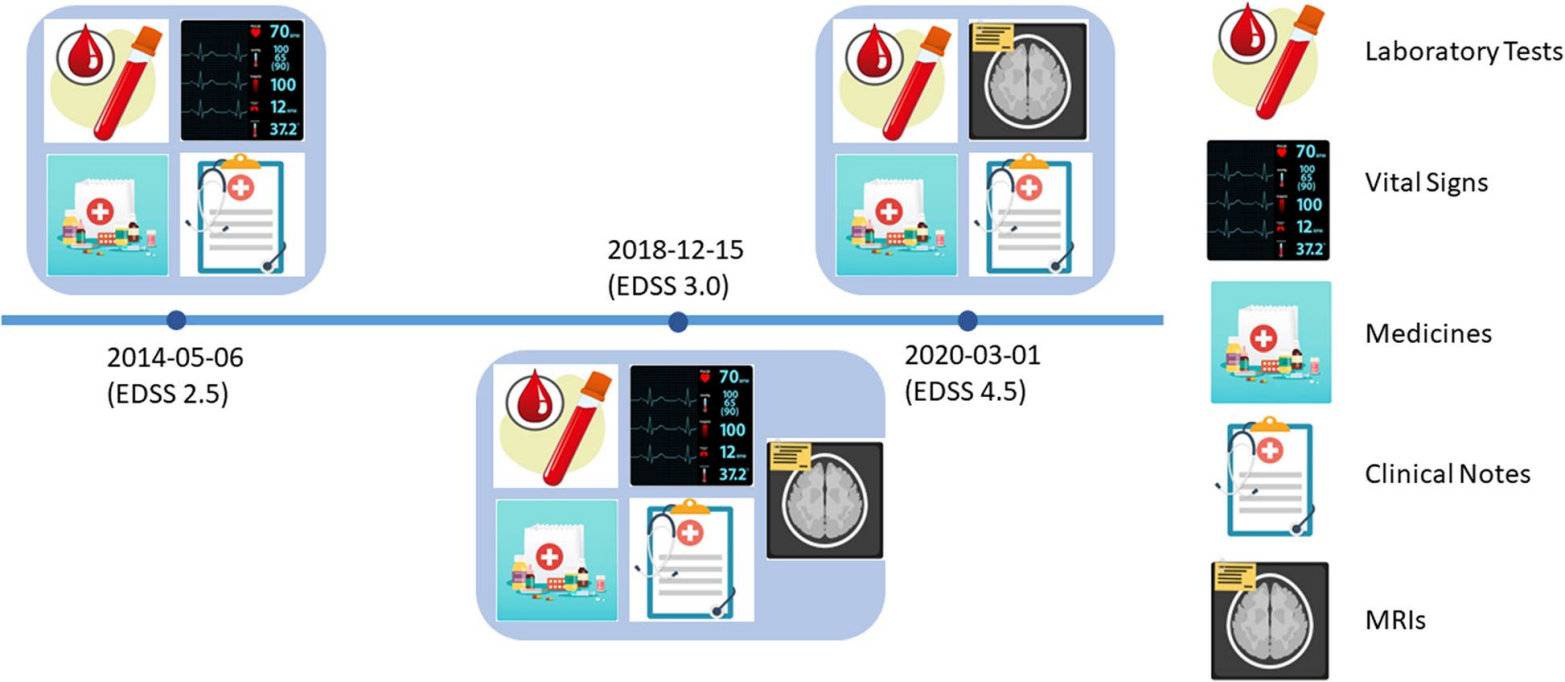
Example: The clinic visits of an example patient and the information (data modality) recorded during each visit

Figure 4 demonstrates an example patient’s three clinic encounters. Note that not all data modalities were observed in each encounter.

### Model architecture

We propose a novel multimodal neural network designed to predict a patient’s EDSS score. The proposed neural network architecture follows an encoder-decoder schema implemented in a sequential structure, augmented with a self-attention module for improved performance and feature extraction capabilities.

**Fig. 5:**
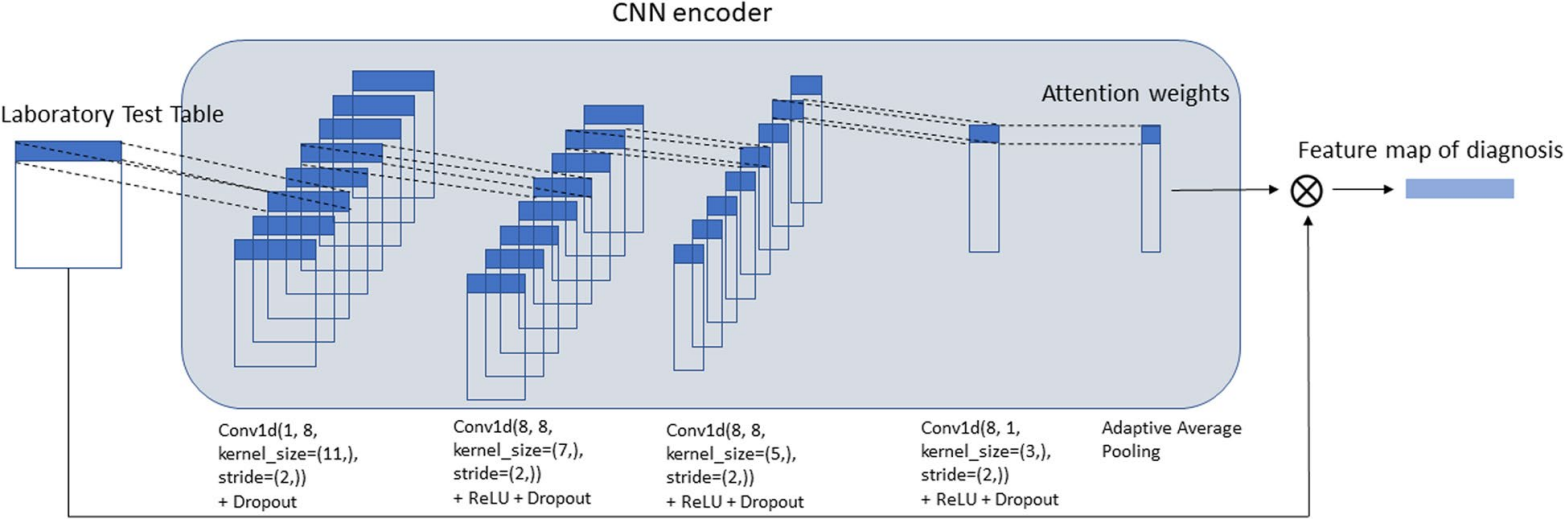
The detailed architecture of one of the encoder channels for processing structured HER data. The figure shows the lab test channel as an example

The objective of the encoder network is to effectively process data from various modalities and map them into dense embeddings within a shared high-dimensional latent space. Distinct encoder neural network architectures are employed for each modality, tailored to their respective learning tasks. For instance, CNNs are utilized for image processing and structured EHR data, while language models are employed for handling clinical notes, see Fig. 5. In the following, we introduce the details of each encoder architecture for each modality.

#### Structured EHR

**Fig. 6:**
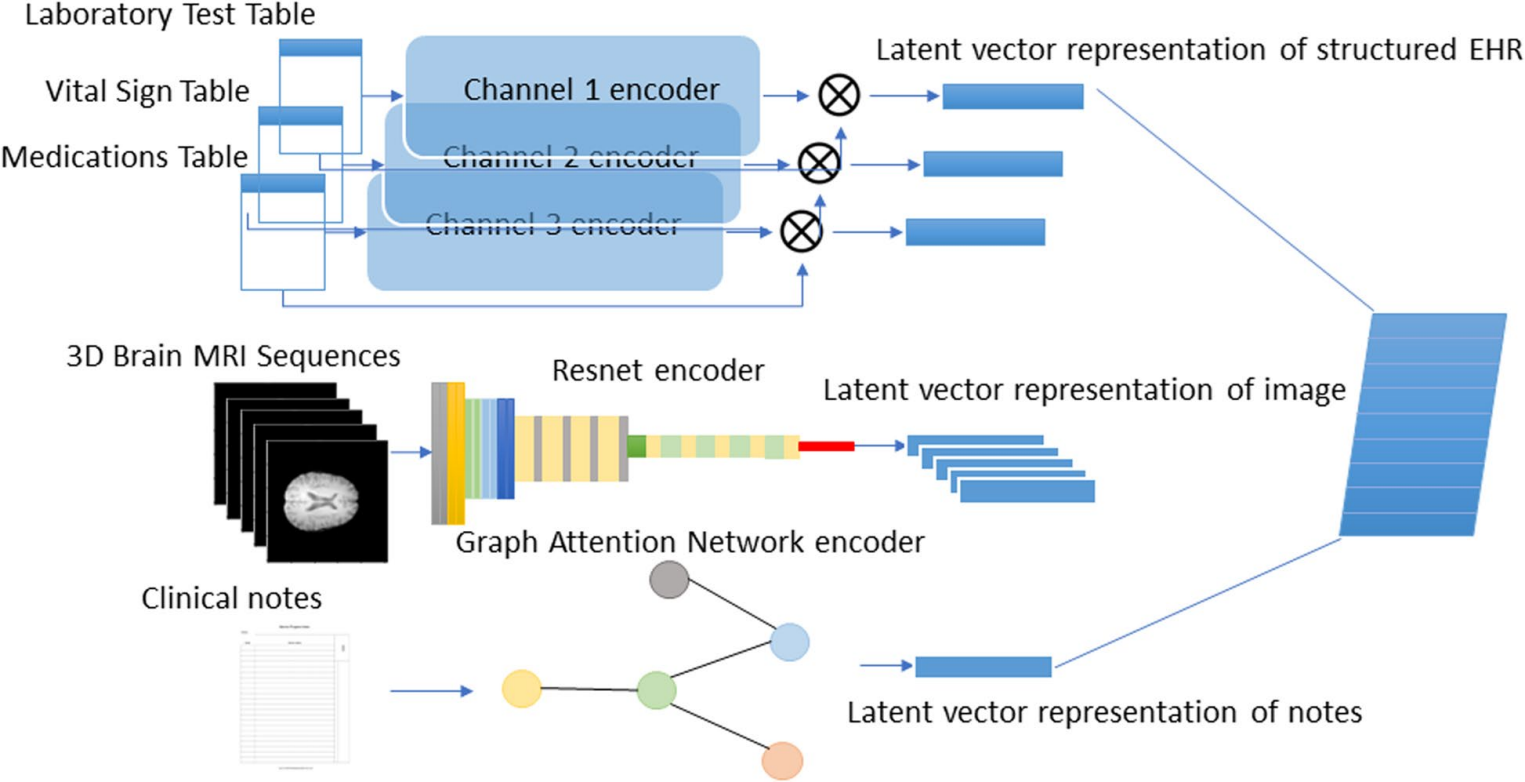
The encoder network for our proposed deep neural network

The encoder network for structured EHR consists of multiple parallel 1-dimensional CNN channels. Each channel within the network follows a homogeneous network structure but incorporates distinct hyperparameters to accommodate EHR tables of varying sizes specific to each patient. This design allows for efficient processing and extraction of meaningful features from the structured EHR data, see Fig. 6.

The structured EHR data of patients comprises multiple tables containing information such as lab tests, vital signs, and medications. These tables are formatted with rows representing observation time points and columns representing specific features. However, the number of rows can vary for different patients and different tables of the same patient, resulting in irregular spacing along the time axis. This irregular spacing introduces heterogeneity in sampling intervals, posing challenges for analysis.

The irregular sampling issue is a typical issue in processing structured EHR data with multiple longitudinal features [20]. Traditional methods such as multiple imputation [21] or Gaussian process-based imputation [22] address this issue by performing imputation. The essential idea is to establish a common regularly spaced time axis for all the features and then imputing missing values at these shared time points. Recent advancements have demonstrated that attention networks offer a more effective solution to this problem, yielding superior performance [23]. This module enables the neural network to adaptively assign distinct attention weights to different time points in a patient’s history, thereby effectively handling the irregularity in the sampling intervals. Specifically, the attention weights are computed for each row (representing a time point) through the application of multiple layers of 1D-CNNs on the feature dimension. This process results in the generation of a single attention weight for each time stamp.

The computed attention weights collectively form an attention vector, which represents the relative importance assigned to different time stamps. By applying this attention vector to the original input data, the network is able to generate a fixed dimensional embedding that remains consistent across all patients. This approach ensures that the neural network is able to capture and leverage relevant temporal patterns and dependencies in the data, enabling more accurate and robust predictions.

In each channel, there are stacked 1D convolution layers, followed by a ReLU activation layer and dropout layers. The number of layers varies depending on the number of features in each table (lab, vital, medication, etc.). For the $i$-th patient, the $k$-th data table ${\varvec{D}}_{k}^{i}$ of dimension ${t}_{k}^{i}\times {f}_{k}$ is fed into the$k$-th channel, where ${t}_{k}$ rows represent the time stamps of clinic visits and ${f}_{k}$ columns represent variables. Note that different EHR tables (laboratory tests, vital signs, medications, etc.) have different ${f}_{k}$ and different patients have different numbers of clinic visits ${t}_{k}^{i}$. Each row of the table is processed through a stack of multiple 1D CNNs (see Fig. 6) and is reduced to a single value (attention weight). The entire table will generate an attention weight vector ${\varvec{\alpha }}_{\varvec{k}}^{\varvec{i}}$ of size ${t}_{k}^{i}\times 1$. The attention weights can be viewed as the weight factor of all ${f}_{k}$ features at different time points. In the following, we omit the patient index $i$.

We multiply the attention vector ${\varvec{\alpha }}_{\varvec{k}}^{ }$ with the input matrix ${\varvec{D}}_{k}$ to get the feature map ek for each table,

$${\varvec{e}}_{k}={\varvec{\alpha }}_{k}^{T}\cdot {\varvec{D}}_{k}$$

where ${\varvec{e}}_{k}$ is the embedding vector of the $k$-th table for a certain patient. Specifically, each element in ${\varvec{e}}_{k}$ is calculated as

$${\varvec{e}}_{k}\left[j\right]=\sum _{m=1}^{{t}_{k}} {\varvec{\alpha }}_{k}\left[m\right]{\varvec{D}}_{k}\left[m,:\right], \text{ for }j=1,\dots ,{f}_{k}$$

and ${\varvec{e}}_{k}$ is of size $1 \times {f}_{k}$.

#### Image embedding

For the encoder channel dedicated to patient MRI images, we employ a different network structure compared to the structured EHR. Specifically, we utilize the ResNet architecture [24] to process the MRI images. Each MRI sequence, namely T1-pre, T1-post, T2, PD, and FLAIR, is individually fed into a corresponding ResNet model. The output of each ResNet model is a fixed-length embedding vector.

#### Clinical notes embedding

The encoder channel dedicated to processing patient clinical notes data employs a Graph Attention Convolution Model (GACN), which takes textual input and generates embeddings for each document [25]. For medical word embeddings, we utilize a pre-trained database trained on PubMed + MIMIC-III [26].

In GACN, the entire document is treated as a word co-occurrence network, where words in the corpus of all patients’ documents serve as graph nodes. Additionally, an extra “document node” is introduced, representing the entire document, and connected to all other nodes. To capture word co-occurrences, a sliding window mechanism is employed, and the resulting co-occurrences are represented as weighted and directed edges in the graph. This ensures that the word order is preserved within the sliding window, while maintaining meaningful semantics and word co-occurrence counts.

The training process of GACN is based on message passing. Specifically, we define $G(V, E)$ as the graphical network, where $V$ represents the set of nodes and $E$ represents the set of edges. Each node $v (\in V)$ constructs a broadcasting message by aggregating the embeddings of its neighboring nodes (using a multi-layer perceptron).

$${\varvec{m}}_{v}^{t+1}={\text{ AGGREGATE }}^{t+1}\left(\left\{{\varvec{h}}_{w}^{t}\mid w\in \mathcal{N}\left(v\right)\right\}\right),$$

which can proceed in a parallel manner using matrix format,

$${\varvec{M}}^{t+1}={\text{MLP}}^{t+1}\left({\varvec{D}}^{-1}\varvec{A}{\varvec{H}}^{t}\right)$$

where ${\varvec{H}}^{t}\in {\varvec{R}}^{n\times d}$ is the $d$-dimensional node features of $n$ nodes and $\varvec{A}\in {\varvec{R}}^{n\times n}$ is the adjacency matrix, and MLP is multiple layer perceptrons neural network.

All nodes update themselves by their own embedding and all messages from their neighbors using a Gated Recurrent Unit (GRU) network,

$${\varvec{h}}_{v}^{t+1}={\text{COMBINE}}^{t+1}\left({\varvec{h}}_{v}^{t},{\varvec{m}}_{v}^{t+1}\right)$$

again, in matrix format,

$${\varvec{H}}^{t+1}=\text{G}\text{R}\text{U}\left({\varvec{H}}^{t},{\varvec{M}}^{t+1}\right).$$

After $T$ steps, a final self-attention read-out layer is used to aggregate all nodes embeddings and output a latent vector to represent the entire document,

$$\begin{array}{c}{\varvec{Y}}^{T}=tan\text{h}\left({\widehat{\varvec{H}}}^{T}{\varvec{W}}_{A}^{T}\right)\\ {\varvec{\beta }}_{i}^{T}=\frac{\text{exp}\left({\varvec{Y}}_{\varvec{i}}^{T}\cdot {\varvec{v}}^{T}\right)}{\sum _{j=1}^{n-1} \text{e}\text{x}\text{p}\left({\varvec{Y}}_{j}^{T}\cdot {\varvec{v}}^{T}\right)}\\ {\varvec{u}}^{T}=\sum _{i=1}^{n-1} {\varvec{\beta }}_{i}^{T}{\widehat{\varvec{H}}}_{i}^{T}\end{array}$$

where ${\widehat{\varvec{H}}}^{T}\in {\varvec{R}}^{n\times d}$ is the final node representation of all $n - 1$ nodes (remove the document node) after $T$ time steps, and ${\varvec{W}}_{A}^{T}$ is the network parameters (a dense layer). Therefore, ${\varvec{u}}^{T}\in {\varvec{R}}^{\varvec{d}}$ would be the final representation of the document, i.e., aggregation of all node features, which will be fed into a classification layer for document classification.

#### Multi-modality data fusion

Multimodal medical data often exhibit inherent logical relationships. For instance, vital signs and laboratory tests contribute to the diagnosis, which in turn determines the appropriate procedures and medications. Some information remains constant over time, such as demographics, while others evolve dynamically.

**Fig. 7:**
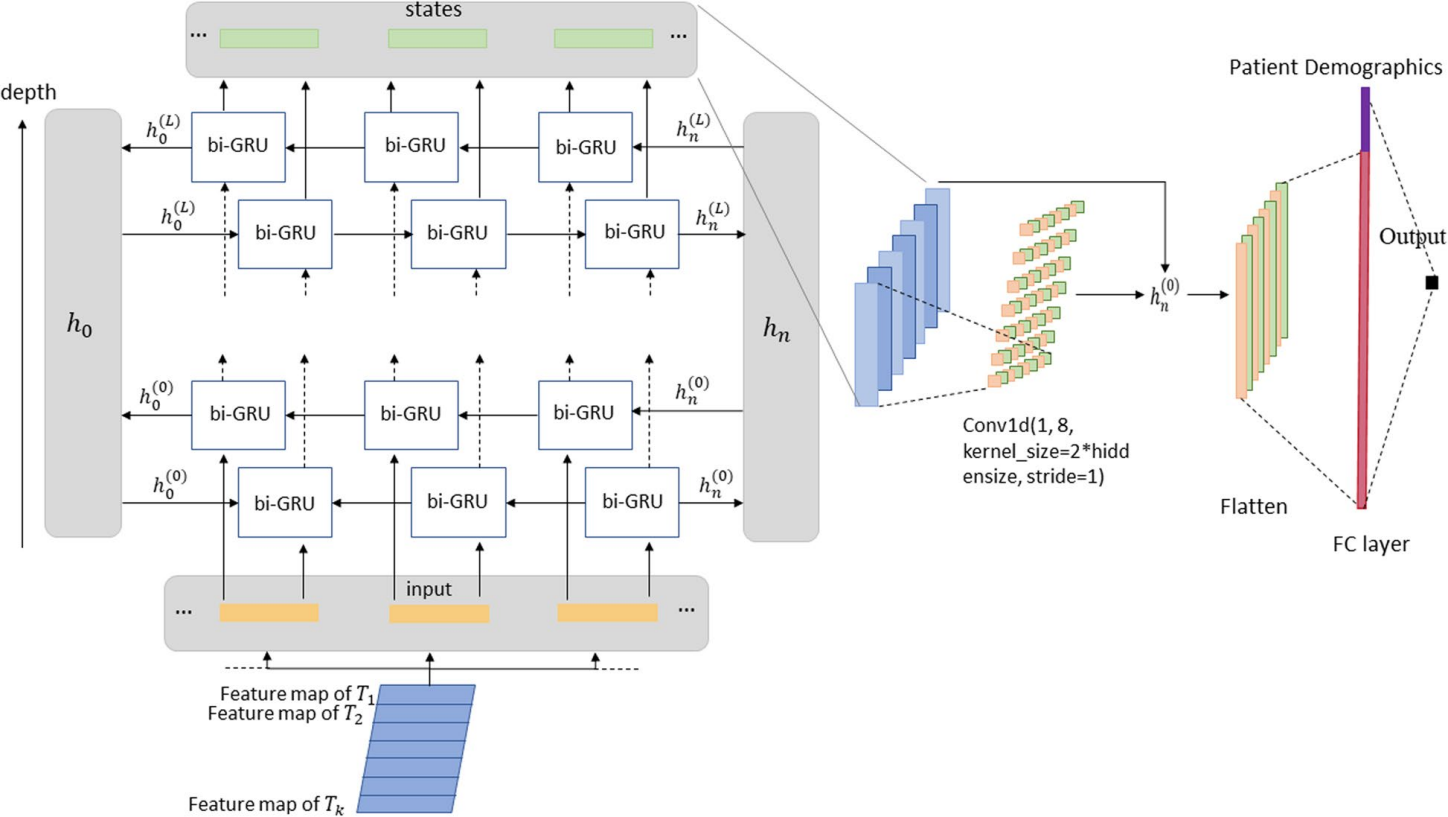
The decoder network for our proposed deep neural network

To take advantage of the intricate interplay among various types of medical information, we have designed a data fusion pipeline that leverages the causal relationships between variables. Vital signs, laboratory tests, and MRI scans leading to the diagnosis, which further influences prescription and procedure decisions, ultimately resulting in medication administration. This pipeline is implemented using a bidirectional GRU-based decoder, facilitating the integration of time-varying information. Therefore, the latent representation vectors obtained from each encoder network channel are combined into a structured matrix $\varvec{E}$ in the above order (illustrated in the left part of Fig. 7). If the lengths of the vectors differ, zero-padding is applied to ensure a consistent matrix format. Each row of the matrix represents a specific modality, enabling the model to capture and learn the interdependencies within the data.

$$\varvec{E}={\left[\text{Z}\text{e}\text{r}\text{o}\text{P}\text{a}\text{d}\text{d}\text{i}\text{n}\text{g}{\left({\varvec{e}}_{1}\right)}^{T},\dots ,\text{ ZeroPadding }{\left({\varvec{e}}_{K}\right)}^{T}\right]}^{T},$$

where $\varvec{E}$ is of dimension $K \times d, d = max({f}_{1}, . . . , {f}_{K}).$

#### Decoder network

The decoder network structure is composed of a stacked bidirectional GRU (Bi-GRU) network with a self-attention module. It takes the feature matrix $\varvec{E}$ as input. The self-attention serves to learn important weights on the state vectors from different data modalities. The Bi-GRU network takes $K$ as the sequence length and $d$ as the input size. We use $\varvec{C}$ to denote the stack of hidden states of all time points, which is of dimension $K \times h$, $h$ = 2 × hiddensize (note that factor 2 comes from the bi-direction network being used).

Each state of the bidirectional GRU network is fed into an attention module, which is 1D convolution layer of multiple output channels. The attention module outputs a vector of attention weights $\varvec{\gamma }$ of length $g$ (hyper-parameter, depending on the output channel of the convolution layer), and

$$\varvec{B}={\left[{\gamma }_{1}^{T},\dots ,{\gamma }_{K}^{T}\right]}^{T}$$

where $\varvec{B}$ is of dimension $K \times g$ denoting the attention matrix. The attention matrix is multiplied with the GRU output,

$$\varvec{O}={\varvec{B}}^{T}\cdot \varvec{C}$$

where $\varvec{O}$ is of dimension $g \times h$. Note that the purpose of this attention layer is to enforce a feature reduction from the high-dimensional GRU outputs to a smaller and more informative lower-dimensional embedding not only for reducing the noise but also for increasing the efficiency of neural network training.

The output matrix $\varvec{O}$ is flattened, and concatenated with the patient demographic data vector $\varvec{d}$, and fed into a fully-connected (FC) layer for prediction,

$$o=\text{F}\text{C}\left(\text{C}\text{o}\text{n}\text{c}\text{a}\text{t}\right(\text{F}\text{l}\text{a}\text{t}\text{t}\text{e}\text{n}\left(\varvec{O}\right),\varvec{d}\left)\right)$$

see Fig. 7.

## Results

The prediction model is implemented using Python and PyTorch, and the training process is conducted on a Tesla A100 graphics card. Before feeding the data into the model, a comprehensive quality check is performed on all modalities. Any low-quality data, such as empty clinical notes or meaningless lab test results, is carefully excluded or removed from the dataset.

To evaluate the model’s performance, a 5-fold cross-validation approach is employed. The dataset of 300 patients is randomly divided into five folds, with each fold used iteratively as the hold-out test set (20%) while the remaining folds serve as the training set (80%). This cross-validation strategy allows for a robust assessment of the model’s predictive capabilities.

The performance of the prediction model in identifying patients with an Expanded EDSS score greater than 4.0 is summarized in Table 3. Multiple data modalities are considered, and their individual and combined contributions to the prediction task are evaluated. It is observed that the utilization of multimodal data inputs generally yields superior performance compared to single-modal inputs. Specifically, the top three performances in terms of AUROC are achieved when utilizing all data modalities (0.8380), combining EHR and clinical notes (0.8078), or combining MRI and clinical notes (0.7988).

**Table 3:** Encoder network parameters (I: input channel size, O: output channel size, K: kernel size, S: stride size, P: padding size, R: (dropout) rate)

	Conv1d	Dropout	Conv1d	ReLU + Dropout	Conv1d	ReLU + Dropout	Pooling
Channel 1 (Lab tests)	I: 1, O: 8, K: 7, S: 2	R: 0.3	I: 8, O: 8, K: 4, S: 2	R: 0.3	I: 8, O: 1, K: 3, S: 2	R: 0.3	Avg.
Channel 2 (Vital Sign Observation)	I: 1, O: 8, K: 3, S: 2	R: 0.3	I: 8, O: 1, K: 2, S: 2	R: 0.3			Avg.
Channel 3 (Medication)	I: 1, O: 8, K: 3, S: 2	R: 0.3	I: 8, O: 1, K: 2, S: 2	R: 0.3			Avg.

Moreover, the degradation in prediction performance resulting from excluding either MRI or EHR data from the input is minimal. This can be reflected on the performance of MRIs & Notes and EHR & Notes in Table 3 which have four out of five metrics falling in the top highest, and three of five in top highest, respectively. This suggests that these modalities provide limited additional information compared to clinical notes when predicting the severity of MS. Notably, if clinical notes are entirely omitted from the input data, the prediction performance drops to 0.7836. Additional information on the model’s performance in predicting other EDSS milestones, such as EDSS > 6 and EDSS > 7, using all data modalities, can be found in Table 4.

**Table 4:** Prediction accuracy performance of using different data modalities for predicting EDSS>4. In each evaluation metric, the top-3 highest scores are highlighted

	AUROC	AUPRC	Sensitivity	Specificity	Accuracy
MRI T1-pre	0.6462 $\pm$ 0.0352	0.2074 ± 0.0145	0.5089 ± 0.0397	0.7679 ± 0.0209	0.6567 ± 0.0300
MRI T1-post	0.6437 $\pm$ 0.0389	0.2027 ± 0.0180	0.5501 ± 0.0390	0.6536 ± 0.0252	0.6697 ± 0.0199
MRI T2	0.7736 ± 0.0268	0.2245 ± 0.0198	0.6834 ± 0.0223	0.7409 ± 0.0398	0.7467 ± 0.0390
MRI FLAIR	0.7945 ± 0.2798	0.3306 ± 0.0309	**0.7689 ± 0.0261**	0.7423 ± 0.0265	0.7423 ± 0.0399
MRI PD	0.5430 ± 0.0401	0.0998 ± 0.0321	**0.7536 ± 0.0218**	0.4862 ± 0.0300	0.5046 ± 0.0399
Clinical Notes	0.7048 ± 0.0365	0.5201 ± 0.0293	0.4632 ± 0.0320	**0.8956 ± 0.0235**	0.4958 ± 0.0301
Structured EHR	0.6589 ± 0.0193	0.3651 ± 0.0265	0.7015 ± 0.0263	0.6587 ± 0.0366	0.6984 ± 0.0265
MRIs & Notes	**0.7988 ± 0.0465**	**0.6321 ± 0.0299**	0.7024 ± 0.0536	**0.7792 ± 0.0563**	**0.7963 ± 0.0422**
MRIs & EHR	0.7836 ± 0.0531	0.4265 ± 0.0323	0.6789 ± 0.0411	0.6875 ± 0.0333	0.6841 ± 0.0523
EHR & Notes	**0.8078 ± 0.0232**	**0.7978 ± 0.0453**	0.7268 ± 0.0435	0.7643 ± 0.0255	**0.8125 ± 0.0353**
MS-BERT( [11])	0.6010 ± 0.0222	0.2064 ± 0.0356	0.3090 ± 0.0265	0.7936 ± 0.0512	0.7788 ± 0.0398
MRI & Notes & EHR	**0.8380 ± 0.0438**	**0.7963 ± 0.0520**	**0.7489 ± 0.0502**	**0.7936 ± 0.0488**	**0.7960 ± 0.0312**

### MRI Images

We introduce five channels to process the MRI sequence, where each channel employs a ResNet structure. The five channels are independent, and each is trained to learn from one sequence (T1-pre, T1-post, T2, FLAIR and PD). All MRI images are bias-corrected, skull-stripped, and registered and the intensity scale is normalized [27]. The following data augmentation is applied during model training. Image intensity normalization and random horizontal and vertical flip were performed both with a probability rate of 0.5. Randomly rotation was performed with a probability of 0.5 by a maximum of ± 0.02 degrees on all three dimensions. Random zoom-out (then resize) was applied to prevent neural networks to take shortcuts by remembering the pixel location instead of learning characteristic lesions areas to make predictions. If a patient performed MRI scans in more than one clinic visit, we use the last scan as it represents the patient’s most recent disease status. Due to the relatively high imbalance of the positive and negative samples, we performed 10-fold re-sampling for the negative training samples during model training.

For each channel, a respective ResNet model is trained on the training dataset, and we select the trained model with the best performance on the validation dataset. Our goal is to learn a latent vector representation of the MRI image instead of performing disease classification at this stage, therefore, the training process is formulated as a metric learning task where each channel’s ResNet is trained to learn an embedding for each MRI sequence of a patient. The triplet margin loss [28] operates directly on embedding distances by promoting the matching point (positive) to the reference point (anchor) and the non-matching point (negative) away from the anchor. The network is trained to learn well-separated embedding vectors for positive and negative patients for downstream decoding networks to perform classification. The triplet margin loss is defined as

$$loss=\sum _{{a}_{i},{n}_{i},{p}_{i}\in \text{b}\text{a}\text{t}\text{c}\text{h} }\text{max}\left(d\left({a}_{i},{p}_{i}\right)-d\left({a}_{i}, {n}_{i}\right)+margin, 0\right)$$

where ${a}_{i},{n}_{i}$ and ${p}_{i}$ are an anchor, positive and negative sample in the batch, respectively. We set the anchor point in our model as a fixed point in the embedding space, therefore, the distance from the positive samples to the anchor is minimized and the distance from the negative samples to the anchor is maximized.

The margin in the triplet margin loss is chosen to be 1.5. The learning rate is set to be 10^−5^ and the batch size is 10. The ResNet in each encoder channel is trained for 500 epochs. Early stopping criteria of not-improving for consecutive 50 epochs on the validation dataset are adopted.

**Fig. 8:**
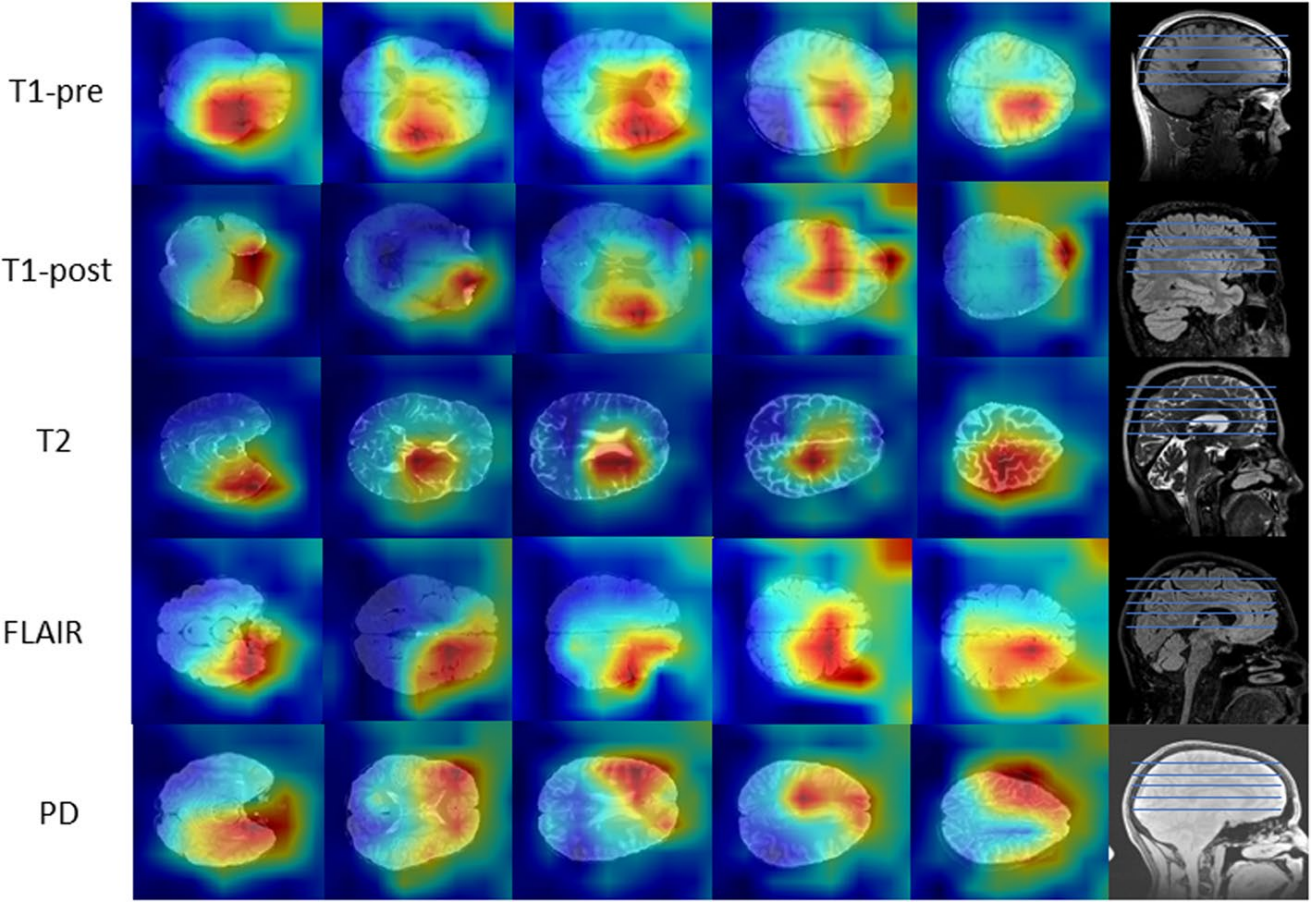
Attention maps for MRI sequences of a sample patient

We leverage the gradient-weighted class activation mapping (Grad-CAM) [29] model to locate and visualize the important regions the ResNet neural network is learning for predicting the target. The Grad-CAM uses flowing gradients of the prediction target into the last convolutional layer of the ResNet to produce a heatmap of the regions according to their contributions to the prediction, see Fig. 8.

### Clinical notes

We preprocess patients’ clinical notes by identifying and then removing all sensitive patient health information that is irrelevant to our prediction task, including the patient and physician’s name, address, phone number, and email address. Similar to the MRI image data, we formulate the embedding generation problem from clinical notes as a metric learning problem, where the message-passing graph neural network is trained to learn meaningful embeddings and their distances between positive and negative samples. Hence, the same loss function (14) is used for this encoder channel.

We set the size of the window to be 10 (covering 10 consecutive words) and the message passing layer to be 2. The hidden side of the GRU network is 64. We trained the graph network with 500 epochs with a batch size of 128, the learning rate of 10^−3^, and early stopping criteria of 50 epochs (no improvements on the validation dataset). We choose the best-performing model on the validation dataset and run it on the test dataset to get the model’s final performance.

### Structured EHR

The patient’s structured EHR consists of tables of 4 categories, the laboratory tests table, the vital signs table, the medications table, and the demographics table. The first 3 categories are in the format number of timestamps × number of features containing the laboratory test results (float), vital sign measurements (float), and medications (0/1 indicators), respectively. All numerical data (non-categorical) was standardized using max-min scalar to the range of 0 to 1. Table 2 shows a pre-selected subset of all the variables from the above 3 categories to be used in our model, based on their observation frequency. Features (lab tests, medications) that were taken by less than 10% of patients were discarded. The categorical features in the demographic table contains race (0/1, one-hot encoded), ethnicity (0/1, one-hot encoded), sex (0/1, male/female), and age (float, min-max normalized). The encoder network consists of 3 channels for each of the first 3 categories and the network parameters are described in Table 5.

**Table 5:** Prediction accuracy performance at different EDSS milestones

	AUROC	AUPRC	Sensitivity	Specificity	Accuracy
MRI & Notes & EHR (EDSS > 4)	0.8380 ± 0.0438	0.7963 ± 0.0520	0.7489 ± 0.0502	0.7936 ± 0.0488	0.7960 ± 0.0312
MRI & Notes & EHR (EDSS > 6)	0.8032 ± 0.0556	0.7012 ± 0.0501	0.8043 ± 0.0454	0.7121 ± 0.0755	0.6720 ± 0.0555
MRI & Notes & EHR (EDSS > 7)	0.8543 ± 0.0572	0.7678 ± 0.0588	0.6777 ± 0.0453	0.7534 ± 0.225	0.7248 ± 0.0377

A patient’s three structured EHR’s embeddings produced by the encoder network will be concatenated with the five MRI image embeddings produced by the ResNet, and together with the clinical note embedding to be fed into the decoder network. In the situation of a patient (a small amount) without MRI or clinical notes, the corresponding embedding will be set to an all-zero vector. In the decoder network, the bidirectional GRU network is set to have 4 layers and hidden size of 512.

**Fig. 9:**
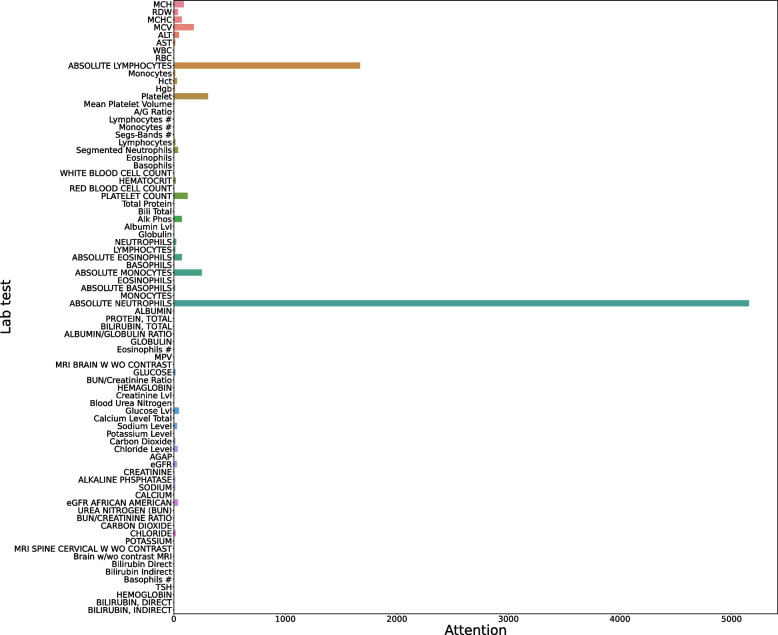
The attention weights for laboratory tests

The self-attention module in the encoder channels corresponding to laboratory tests, vital signs, and medications can provide insights into feature importance. The importance of a feature represents how much the feature is being relied on making correct predictions. Figure 9 illustrates the importance of laboratory features evaluated on the test set of patients. Larger value indicates higher feature importance. From the figure, we observe that the top three important features for all patients are “Absolute Neutrophils”, “Absolute Lymphocytes”, and “Platelet”.

**Fig. 10:**
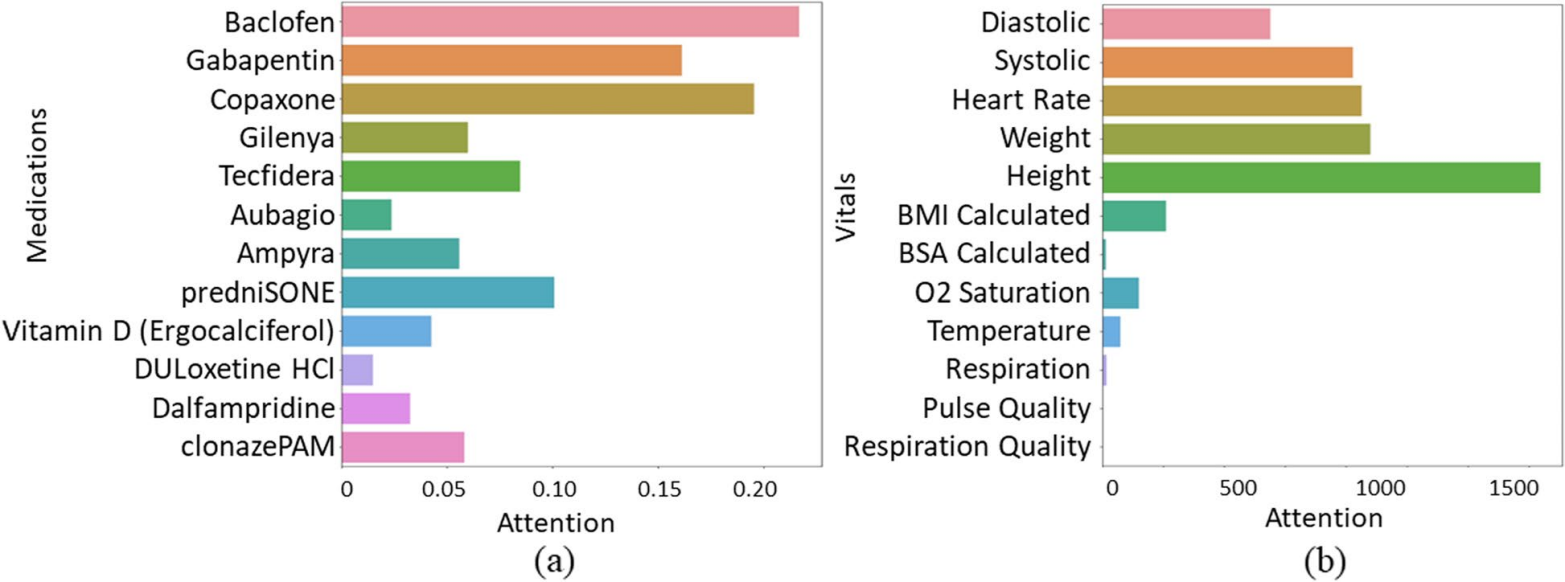
The attention weights for (**a**) medications and (**b**) vital signs

Similarly, Fig. 10 depicts the feature importance for vital signs and medications. Our algorithm identifies certain medications such as “Baclofen 10 MG Oral Tablet”, “Gabapentin 300 MG Oral Capsule”, and “predniSONE 50 MG Oral Tablet” as having high importance, as they are commonly used to treat MS symptoms. Regarding vital signs, features such as “Temperature”, “Respiration”, “Pulse Quality”, and “Respiration Quality” are generally regarded as less critical indicators for predicting the severity of MS in the clinical consensus.

These findings provide valuable insights into the relevance of specific features for the prediction of MS severity, aiding in understanding the underlying factors and potential treatment options.

## Discussion

In this study, we propose a multimodal deep neural network approach that combines EHR and neuroimaging data to address the prediction of MS disease severity. By leveraging diverse sources of information such as laboratory tests, vital signs, medications, neuroimaging data, and clinical notes, our model aims to provide accurate predictions of the EDSS score, a widely used metric for evaluating MS disease severity.

The study focuses on three EDSS milestones EDSS 4.0, 6.0 and 7.0 since they are widely accepted as critical transition points between MS stages. For example, Confavreux et al. used the above milestones to study the effect of relapses on the progression of irreversible disability [30]. The same milestones have also been used to study the contribution of relapses to worsening disability and evaluate the MS therapies’ effect on delaying the disability accumulation [31]. A Sweden research group studied whether the risk of reaching the above disability milestones in MS has changed over the last decade [32]. Rzepiński et al. used the EDSS milestones to explore early clinical features of MS and how they affect patients’ long-term disability progression [33]. The same milestones were also used to study how these factors affect the time to transition from relapsing-remitting MS (RRMS) to secondary progressive MS (SPMS).

While MRI images and clinical notes have been recognized as valuable sources of diagnostic information for MS, the role of laboratory test results in predicting the severity of the disease remains uncertain. This study aims to contribute to the understanding of this matter from an engineering perspective. Conversely, previous research has indicated that both MRI data and certain laboratory test results can provide meaningful insights into MS disease severity. Notably, studies have demonstrated a strong correlation between the thickness of cortical and deep grey matter in MRI images and the severity of MS, underscoring the informative nature of MRI data in predicting disease progression [34, 35]. Some laboratory tests were also documented as playing an important role in this regard, such as the cerebrospinal fluid (CSF) [36, 37], and serum neurofilament light chain (nFl) [38].

The results show that despite the many publications, conventional MRI contains relatively less information about MS severity compared to other data modalities. However, T2 and Flair MRI performed relatively better than other MRI sequences. Clinical notes were well-documented to be used for the prediction of EDSS, which has been re-verified in our experiment as the relatively not good performance of using MRIs, or EHR, or MRIs & EHR were all improved when clinical notes were added to the input. A re-examination of the data reveals a reasonable explanation that the clinical notes contain rich patient general disease information including patient status, medical procedures, and treatment information, which implicitly and partially embeds information from the EHR data and MRI images.

For MRI image processing, alternatively, other variants of ResNet [39] can also be utilized as embedding learning networks in our task. However, our experimental findings indicate that employing different network structures for the MRI sequences only leads to marginal improvements in prediction performance. This can be attributed to two reasons. Firstly, the inherent capabilities of the ResNet model enable it to effectively capture essential features within the MRI images, thereby generating diverse embeddings for positive and negative patients. Secondly, considering that the MRI data represents only a subset of the overall input multimodal data, the impact of ResNet variations on the final prediction outcome is diluted by the presence of other data modalities.

There are a few future research directions for this study. First, an equally interesting research question is to predict a patient’s MS disease progression rate. This is because having an EDSS of 4.0 at the age of 65 and a disease duration of 40 years would mean a relatively benign disease but having an EDSS score of 4.0 only after 5 years of MS diagnosis is considered as “aggressive” MS. Moreover, the severity of MS can be seen as a relative concept instead of an absolute one. The severity of MS should be studied based on an understanding of the “natural” disease progression, and it varies in terms of many factors (e.g. sex, disease duration, lesion load, atrophy, etc.). Limited by the data size and commonly agreed on criteria to distinguish the “aggressive” cases from the rest, we focus on developing a tool to predict EDSS milestones now and leave the decision of MS severity to MS specialists by jointly considering all the above factors. In addition, this problem itself is quite an interesting research problem and could potentially be studied using survival analysis methods, the results will have a high impact on the prevention of rapid disease progression through early intervention.

The second is the limitation of the imaging data. While random rotation of MRI scans (a data augmentation technique used to train ResNet on the MRI sequences) helps generalizability, the use of only one scanner for all datasets makes it difficult to infer if the model would work in the same way when introduced to new images from a different scanner. Therefore, our work serves as a proof-of-concept regarding this question. Ideally, more data (especially data from external sources) needs to be collaboratively collected to verify the inclusion of MRI potentially has a positive impact on a multi-modal model.

Thirdly, the study was conducted on a cohort of 300 MS patients from a local academic medical center. An important future research direction is to evaluate the generalizability of the proposed model to other institutions. The result replicability should be checked from two perspectives, the first is the prediction accuracy with or without model re-training, i.e., model generalizability; and the second is if the ranking of importance for different data modalities is the same in general, for example, MRI images and clinical notes contains more signals compared to the structured EHR. If the results in this study are verified, it may serve as a cost-effective study recommending which electronic health information should be collected to reach maximum prediction accuracy. To address the issue of limited size of the dataset, collaborative studies are encouraged that involve pooling datasets from various sites. Such an approach could leverage federated learning with secure data sharing mechanisms to facilitate joint investigations. This not only has the potential to enrich our dataset but also aligns with the emerging field of Multimodal Federated Learning, offering an exciting avenue for future research.

Another compelling research question from a technical standpoint revolves around the utilization of time windows for averaging observations. As discussed, this technique proves valuable in reducing the size of longitudinal data while retaining essential temporal information. However, there exist more advanced methods for handling long sequences of temporal data. Although not the primary focus of this study, it is worth mentioning some notable techniques, such as data resampling (subsampling) and the application of deep neural networks capable of handling longer data sequences without encountering issues like the vanishing gradient problem, such as the use of transformer models.

## Conclusion

The study focuses on predicting patients’ MS severity three years in the future by using current and historical, and multimodal medical information, with the goal of developing an AI-based patient disease status evaluation tool to exceed human capabilities.

This research represents an initial exploration in integrating multiple data modalities for predicting MS severity, while also assessing the effectiveness of each modality in this prediction task. Our experimental results highlight the significant contributions of brain MRI images and clinical notes as the most informative modalities for predicting MS severity, while structured EHR data demonstrates relatively limited relevance to this specific prediction objective. By integrating and analyzing multimodal data, our approach aims to improve the understanding of MS disease progression and provide valuable insights for clinical decision-making and treatment planning.

### Supplementary Information

**Additional file 1: Table S1. **Performance comparison between the proposed method (attention) with other missing data imputation methods on predicting EDSS > 4.**Table S2. **Performance comparison between the proposed multimodal deep learning method with other multimodal data fusion techniques on predicting EDSS>4.

## Data Availability

The data that support the findings of this study are available on request from the corresponding author SS. The data are not publicly available due to their containing information that could compromise the privacy of research participants. Code is publicly available on Github: https://github.com/anotherkaizhang/MS.

## Abbreviations

MS: Multiple sclerosis
EDSS: Expanded disability status scale
EHR: Electronic health records
AUROC: Area under the receiver operating characteristic curve
AUPRC: Area under the precision-recall curve
MRI: Magnetic resonance imaging
SD: Standard deviation
PD: Proton density
GRU: Gated recurrent unit
Grad-CAM: Gradient-weighted class activation mapping
BCE: Binary cross entropy
