# Supplementary material for: Predicting multiple sclerosis severity with multimodal deep neural networks
Source: BMC Med Inform Decis Mak. 2023 Nov 9;23:255. doi: 10.1186/s12911-023-02354-6 (PMC10634041; doi:10.1186/s12911-023-02354-6)
Supplement: Supplementary file 1 — Additional file 1: Table S1. Performance comparison between the proposed method (attention) with other missing data imputation methods on predicting EDSS > 4.Table S2. Performance comparison between the proposed multimodal deep learning method with other multimodal data fusion techniques on predicting EDSS>4. [file 12911_2023_2354_MOESM1_ESM.docx]

**Supplementary**

1. **Comparison experiment of different missing data imputation techniques**

When handling EHR data with irregular sampling, it is common practice to establish a uniform time grid to address missing observations. These missing observations on the grid are typically treated as missing values, and various imputation methods, such as filling with zeros or averages, forward filling, or multiple imputation, are employed to estimate these missing values. The proposed model takes a different approach by treating the observed data as continuous observations, disregarding the time intervals between observations. In other words, the time intervals between two observed time stamps can vary in length, but the proposed method makes no attempts to impute values between any two time points. As an ablation study to compare different imputation methods with our method, we present the performance results in Table S1.

Table S1. Performance comparison between the proposed method (attention) with other missing data imputation methods on predicting EDSS > 4.

|  | **AUROC** | **AUPRC** | **Sensitivity** | **Specificity** | **Accuracy** |
| --- | --- | --- | --- | --- | --- |
|  |  |  |  |  |  |
| Fill with zeros | 0.7586 ± 0.0784 | 0.7844 ± 0.0534 | 0.7053 ± 0.0348 | 0.7465± 0.0645 | 0.7058 ± 0.0344 |
|  |  |  |  |  |  |
| Forward filling | 0.7678 ± 0.0434 | 0.7961 ± 0.0443 | 0.7155 ± 0.0454 | 0.7053 ± 0.0537 | 0.7254 ± 0.0444 |
|  |  |  |  |  |  |
| Fill with mean | 0.7254 ± 0.0453 | 0.7782 ± 0.0544 | 0.7301 ± 0.0230 | 0.7912 ± 0.0530 | 0.7444 ± 0.0525 |
|  |  |  |  |  |  |
| Our method (attention) | 0.8380 ± 0.0438 | 0.7963 ± 0.0520 | 0.7489 ± 0.0502 | 0.7936 ± 0.0488 | 0.7960 ± 0.0312 |
|  |  |  |  |  |  |

1. **Comparison experiment of different multimodal data fusion techniques**

Modality fusion is one of the important research directions in multimodal learning. In multimodal deep learning fusion, early fusion and late fusion are two different approaches for combining information from multiple modalities. Early fusion is also known as the feature-level or input-level fusion. It merges all modalities at the beginning of the network architecture by combining the raw or preprocessed data into a single input representation. For example, if the modalities are images and text, early fusion would concatenate the image features and text features into a single feature vector or tensor. This combined representation is then fed into the network for predictions. On the other hand, late fusion (decision-level or output-level fusion) is the idea of processing each modality separately through individual networks, and then combining their outputs at a later stage. The predictions from these individual models are then combined using various techniques such as embedding concatenation, averaging, voting, or weighted fusion.

Both methods have their advantages and trade-offs. Early fusion allows the model to directly learn joint representations from multiple modalities but may face challenges in handling diverse and heterogeneous data, and usually needs a preprocessing step to extract useful features to form the input data. Late fusion, on the other hand, allows flexibility in processing individual modalities but may not fully exploit the interactions between modalities because the fusion happens right before the final decision network layer.

The proposed model lies in between the early fusion and late fusion methods. By fusing all modalities embeddings in the intermediate stage of the neural network, the model harmonizes both methods’ advantages. We compare our multimodal deep learning model with the late fusion, and various ensemble methods. For late fusion, we concatenate all EHR tables into one table and feed it into the CNN encoder to generate an embedding and use Resnet and Graph Attention Network encoder to process the MRI data and notes into embeddings. Following the late fusion method, we concatenate the three embeddings into a vector and feed it into a classification layer (Multilayer Perceptrons, MLP) for classification. For the ensemble model, we use the same encoders for different modalities but build prediction models separately, and ensemble the prediction result. For the ensemble methods, we tried soft voting (taking the average of the predicted probability after calibration) and hard voting (taking the majority of the predicted 0 or 1 for the binary classification task). The result is shown in Table S2.

Table S2. Performance comparison between the proposed multimodal deep learning method with other multimodal data fusion techniques on predicting EDSS>4.

|  | **AUROC** | **AUPRC** | **Sensitivity** | **Specificity** | **Accuracy** |
| --- | --- | --- | --- | --- | --- |
| Late fusion (feature fusion) | 0.7356 ±0.0231 | 0.7272 ±0.0341 | 0.6784 ±0.0451 | 0.7327 ±0.0444 | 0.6304 ±0.0404 |
| Ensemble model (Soft voting) | 0.8132 ±0.0565 | 0.7526 ±0.0365 | 0.8056 ±0.0514 | 0.6335 ±0.0545 | 0.8044 ±0.0365 |
| Ensemble model (Hard voting) | 0.7770 ±0.0385 | 0.7874 ±0.0564 | 0.6350 ±0.0614 | 0.8334 ±0.0641 | 0.7798 ±0.0323 |
| Our method | 0.8380 ±0.0438 | 0.7963 ±0.0520 | 0.7489 ±0.0502 | 0.7936 ±0.0488 | 0.7960 ±0.0312 |
